# *Addiction Science & Clinical Practice*: a new partnership with the Grayken Center for Addiction at Boston Medical Center to usher in our next phase

**DOI:** 10.1186/s13722-024-00499-x

**Published:** 2024-09-13

**Authors:** Katherine E. Calver, Jeffrey H. Samet, Emily C. Williams

**Affiliations:** 1 Boston Medical Center, Boston, MA USA; 2 Department of Medicine, Section of General Internal Medicine, Boston Medical Center, Boston University Chobanian and Avedisian School of Medicine, Boston University School of Public Health, Boston, MA USA; 3 Department of Health Systems and Population Health, School of Public Health, University of Washington; Center of Innovation for Veteran-Centered and Value-Driven Care, Health Services Research & Development, VA Puget Sound, Seattle, WA USA

In 2022, we announced [1] that our masthead was growing, with Dr Emily Williams stepping into the role of *Addiction Science & Clinical Practice*’s co-Editor-in-Chief (EIC), leading alongside co-EIC Dr Jeffrey Samet. We are now pleased to announce that *ASCP* enters this next phase with a new partner: the Grayken Center for Addiction at Boston Medical Center. As the joint owner of the journal (together with our publisher, Springer Nature/BMC), the Grayken Center for Addiction will ensure the longevity of *ASCP*, while preserving its editorial independence.

Since its transition from a National Institute on Drug Abuse journal in 2011 to its current form as an open access publication of Springer Nature/BMC, *ASCP* has enjoyed over a decade of continuous growth. We believe that the journal’s longevity is rooted in the importance of its core mission: to publish research that seeks to address the quality of care for people with unhealthy substance use across a spectrum of clinical settings. In 2012, introducing the rebranded journal in an editorial, “Science to improve care for people affected by unhealthy alcohol and other drug use,” then-co-EICs Drs Richard Saitz and Samet wrote: “In the health-care sector, attention to unhealthy substance use cannot be limited to highly specialized care settings; most patients with these conditions appear in general health settings where such problems are all too often ignored.” [2]. The editorial called for an increase in the quality and effectiveness of screening and treatment for people in primary care and other non-specialty settings, and for greater scrutiny of the effects of unhealthy substance use below the highest thresholds. More than ten years on, we remain dedicated to this mission while also building on it to advance equity in identification and evidence-based treatment of addictions and address structures that produce inequity [1].

We could not have hoped for a better partner than the Grayken Center for Addiction at Boston Medical Center to shepherd this work forward. Founded in 2017, the Grayken Center for Addiction is a national resource for substance use disorder treatment and education, research, advocacy, and thought leadership. As part of Boston Medical Center, the region’s safety net hospital, the Grayken Center for Addiction is driving innovation in substance use treatment, offering patients with varying identities and unique lived experiences low-barrier, patient-centered programs to address unhealthy substance use across a variety of settings, and provide harm-reduction initiatives and wrap-around care.

Under the leadership of Medical Director Dr Miriam Komaromy, the Grayken Center for Addiction’s treatment programming is focused on addressing known gaps in substance use care with evidence-based approaches. The program’s Rapid ACCESS Recovery Coaching program, and Faster Paths medication bridge clinic provide swift evaluation, support, and referral to a number of low-barrier substance use treatment options within Boston Medical Center and the community. Likewise, Project ASSERT provides screening, brief intervention, and counseling to patients in the Emergency Department, ensuring that this critical touchpoint with the healthcare system is supportive to people who may benefit from substance use treatment or harm-reduction services.

The Grayken Center for Addiction also specializes in substance use treatment for special populations, often those most vulnerable to the harms of substance use. A variety of specialized substance use disorder programs serve adolescents and young adults, pregnant people, parents of infants, and people who are unhoused.

The best-known program under the Grayken umbrella is the Office-Based Addiction Treatment (OBAT) program, led by Colleen Labelle MSN, RN-BC, CARN, who also leads the Grayken Center’s Training and Technical Assistance (TTA) Program. BMC’s OBAT program is the largest program providing pharmacotherapy for opioid and alcohol use disorders in New England, serving over 800 patients. OBAT providers integrate substance use treatment into primary care, ensuring that patients’ medical needs are addressed and care coordinated by a single team. The OBAT program is a nationally recognized leader upon which similar programs in outpatient settings across the country have been modeled, and the OBAT model has been replicated in a wide variety of healthcare institutions.

As the Grayken Center for Addiction leads the way in innovative substance use treatment programs and research, it also serves as a center for advocacy and education. Its substance use policy-related efforts have been influential in Boston, the Commonwealth of Massachusetts, and on a national level. The Grayken Center for Addiction’s Addiction Medicine Fellowship program is nationally recognized for its excellence in training the next generation of addiction medicine physicians. The Center’s Addiction Nursing Fellowship is a unique resource supporting the critical role that nurses play in treatment of substance use disorders.

Importantly, while *ASCP* and the Grayken Center for Addiction are aligned in their missions and dedication to improving substance use treatment, treatment access, and quality of care, the journal will retain its editorial independence and receive no editorial input from the Grayken Center for Addiction or its representatives. Our peer and internal review processes remain as robust as ever, and the recent onboarding of several new Associate Editors has only strengthened these processes.

While our editorial team and processes remain independent, the work of the Grayken Center for Addiction is not absent from the pages of *ASCP*. The first submissions to a new, ongoing series—The Grayken Lessons Case Conference Reports—have already been published. These reports describe cases in substance use treatment recently presented by addiction medicine fellows as a part of the Grayken Center for Addiction’s regular case conference series and provide critical insight about addiction treatment approaches. Each include expert commentary from the physicians, nurses, and other providers involved in each case. Over time, these case conference reports will constitute a growing repository of expertise and insight into lessons learned from treating complex patients with substance use problems. Submissions to the Grayken Lessons series are subject to non-biased external and internal peer review, like any other manuscript.

As we step into this next phase, *ASCP* remains committed to the principle that substance use treatment should be based on the best available evidence and accessible outside of specialty care settings. Over a decade after the publication of “Science to improve care for people affected by unhealthy alcohol and other drug use,” [2] there indeed remains much to learn about engaging and retaining people with unhealthy substance use in care and addressing barriers to quality care rooted in stigma and bias, especially among minoritized groups. With the support of and partnership with Boston Medical Center’s Grayken Center for Addiction—a pioneer in substance use treatment, research, and education—we continue and expand our mission.
